# Use of a Novel Artificial Intelligence Platform on Mobile Devices to Assess Dosing Compliance in a Phase 2 Clinical Trial in Subjects With Schizophrenia

**DOI:** 10.2196/mhealth.7030

**Published:** 2017-02-21

**Authors:** Earle E Bain, Laura Shafner, David P Walling, Ahmed A Othman, Christy Chuang-Stein, John Hinkle, Adam Hanina

**Affiliations:** ^1^ AbbVie Inc. North Chicago, IL United States; ^2^ AiCure, LLC New York, NY United States; ^3^ CNS Network, LLC Garden Grove, CA United States; ^4^ Chuang-Stein Consulting Kalamazoo, MI United States; ^5^ EarlyPhase Sciences, Inc. Cary, NC United States

**Keywords:** medication adherence, artificial intelligence, clinical trials as topic

## Abstract

**Background:**

Accurately monitoring and collecting drug adherence data can allow for better understanding and interpretation of the outcomes of clinical trials. Most clinical trials use a combination of pill counts and self-reported data to measure drug adherence, despite the drawbacks of relying on these types of indirect measures. It is assumed that doses are taken, but the exact timing of these events is often incomplete and imprecise.

**Objective:**

The objective of this pilot study was to evaluate the use of a novel artificial intelligence (AI) platform (AiCure) on mobile devices for measuring medication adherence, compared with modified directly observed therapy (mDOT) in a substudy of a Phase 2 trial of the α7 nicotinic receptor agonist (ABT-126) in subjects with schizophrenia.

**Methods:**

AI platform generated adherence measures were compared with adherence inferred from drug concentration measurements.

**Results:**

The mean cumulative pharmacokinetic adherence over 24 weeks was 89.7% (standard deviation [SD] 24.92) for subjects receiving ABT-126 who were monitored using the AI platform, compared with 71.9% (SD 39.81) for subjects receiving ABT-126 who were monitored by mDOT. The difference was 17.9% (95% CI -2 to 37.7; *P*=.08).

**Conclusions:**

Using drug levels, this substudy demonstrates the potential of AI platforms to increase adherence, rapidly detect nonadherence, and predict future nonadherence. Subjects monitored using the AI platform demonstrated a percentage change in adherence of 25% over the mDOT group. Subjects were able to use the technology successfully for up to 6 months in an ambulatory setting with early termination rates that are comparable to subjects outside of the substudy.

**Trial Registration:**

ClinicalTrials.gov NCT01655680 https://clinicaltrials.gov/ct2/show/NCT01655680?term=NCT01655680

## Introduction

Accurately monitoring and collecting drug adherence data can allow for better understanding and interpretation of the outcomes of clinical trials [1-3]. The advent of electronic monitoring has generated a wealth of published data on the value of analyzing drug adherence and using this information as an explanatory variable in itself [4-6]. In order to understand the dose-response relationship of an investigational drug, or understand factors contributing to intersubject variability in response to a drug, it is imperative to properly capture and understand the dosing history (ie, actual doses taken, missed doses, late doses). To ignore these variations in adherence is a “lost opportunity if not a scientific lapse” [4] in both clinical research and in population health.

### Monitoring Methods in Clinical Research

Most clinical trials use a combination of pill counts and self-reported data to measure drug adherence, despite the drawbacks of relying on these types of indirect measures. It is assumed that doses are taken, but the exact timing of these events is often incomplete and imprecise. Pill counts have frequently been shown to underestimate poor adherence and nonadherence [1,7-13].

A recent study questioned the utility of pill count data when compared with pharmacokinetic data [2]. For the 1765 subjects receiving active drug in 8 Phase 2 or later psychiatric trials conducted between 2001 and 2011, the estimated nonadherence rates of 12.8-39.2% from the pharmacokinetic data (with nonadherence defined as >50% of pharmacokinetic samples below the limit of quantification for the study drug in plasma) proved far higher than the nonadherence rates of 0.0-5.1% estimated by pill counts in 5 of the 8 studies [2].

The advent of electronic monitoring packaging (EMP) allowed for more reliable collection of adherence data, giving researchers the ability to collect the date and time stamp of each bottle/package opening (frequently in real time), and providing patients with some reminders and feedback. The short duration of many of the trials that implemented EMP (and the variability in quality of the study designs) in a systematic review of 37 studies using forms of EMP precluded definitive assessment of their effect on adherence [14]. One limitation of EMPs is that they do not verify drug administration. Meticulously removing pills from pill bottles led researchers to prematurely halt a large human immunodeficiency virus (HIV) prevention trial (VOICE) for lack of efficacy, with data suggesting that approximately 70% of the female participants in the study had no measurable tenofovir blood concentrations (the main study drug under investigation) despite approximately 90% of these patients claiming to be adherent [15,16]. More reliable measures tend to be hardware-based and require changes to the drug manufacturing process itself, incurring high costs and operational challenges [17].

Due to its ability to ensure treatment adherence, directly observed therapy (DOT) has been used for decades, both to measure and maximize adherence for treatment of tuberculosis infections and antiretroviral therapies [18-21], and to ensure ingestion in inpatient settings or in early‑phase clinical trials when subjects are dosed in the clinic. However, for trials conducted in outpatient populations, the cost and logistical complexity of administering DOT forces clinical trials to switch to less intensive monitoring, despite the continued and largely unmeasured risk of nonadherence [22].

### Artificial Intelligence Platform

**Figure 1:**
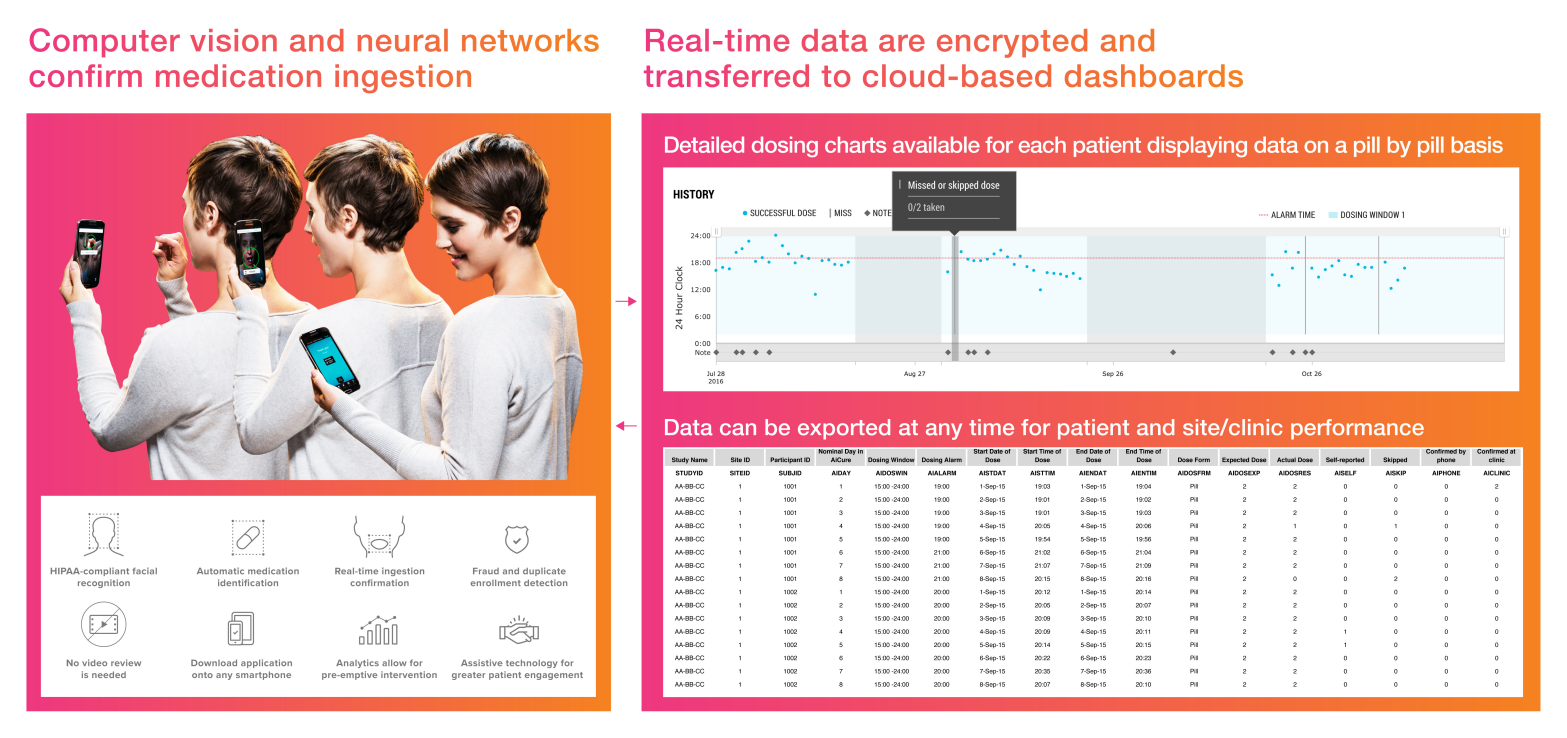
Artificial intelligence platform.

The artificial intelligence (AI) platform AiCure (New York, NY) uses AI to visually confirm medication ingestion (Figure 1) via software that can be downloaded as an app on any mobile device. Using facial recognition and computer vision, software algorithms identify the patient, the drug, and confirm ingestion. Date and time stamps are collected for each individual pill. Adherence data fall into the following 6 categories: (1) visual confirmation of ingestion using the AI platform app, (2) self-reported dose via the *self-report* button in the app (no visual confirmation), (3) self-reported dose over the phone to the study coordinator, (4) missed dose, (5) skipped dose, and (6) dose taken in clinic. Encrypted data for each dosing administration are sent to cloud‑based dashboards for real-time monitoring and intervention, with suspicious activity, duplicate enrollment, or incorrect usage triggering alerts. Study subjects were provisioned a smartphone with the AI app predownloaded to monitor study drug compliance. The AI app was installed with Health Insurance Portability and Accountability Act-compliant AI software.

The present report describes medication adherence results from an exploratory pilot substudy, using the AI platform compared with modified DOT (mDOT) 3 times per week during a clinical study (Study M10-855 [ClinicalTrials.gov NCT01655680]) of an investigational adjunctive oral medication (ABT-126) that was evaluated for treatment of cognitive impairment in patients with schizophrenia. The objectives of this exploratory pilot substudy were to evaluate the AI platform as a real‑time monitoring method for study drug adherence, and to examine the feasibility of using the platform in a 6‑month Phase 2 schizophrenia study.

## Methods

Study M10-855 (AbbVie Inc.) was a Phase 2, multicenter, randomized, double-blind, placebo‑controlled, dose-ranging, parallel-group, 24‑week study of the safety and efficacy of an investigational adjunctive treatment (ABT-126) for the treatment of cognitive deficits in nonsmoking subjects with schizophrenia who were clinically stable. The study was conducted from May 2012 to July 2014 at a total of 31 sites in the United States, 20 sites in Russia, and 7 sites in the United Kingdom. Overall, 431 subjects were randomized to 25, 50, or 75 milligrams (mg) ABT-126 or matching placebo administered as 3 capsules once daily in the morning. All subjects provided informed consent prior to any study procedures.

Adherence was measured by review of returned study drug blister cards. Subjects with less than 70% adherence received intense counseling on the importance of adherence, and could also be withdrawn from the study. In addition, a later amendment of the protocol included an optional adherence program (AI substudy) for US sites. Ten of the 31 US sites agreed to participate. In addition to the blister cards, subjects at these sites were asked to choose between the AI platform and mDOT as a further adherence measure. mDOT required study staff (or a third party) to observe and record study drug adherence at least 3 times per week. Subjects monitored by the AI platform were assigned a device with the AI app downloaded. Adherence data from the AI substudy were not entered into the clinical study database. Sites participating in the AI substudy continued to record adherence based on returned blister cards at each visit.

Pharmacokinetic blood samples for the analysis of ABT-126 plasma concentrations were collected at weeks 2, 4, 6, 10, 12, 16, 18, 22, and 24. At weeks 2 and 4, the pharmacokinetic samples were collected prior to dosing at the site on the week 2 and week 4 visit days. At weeks 6, 12, 18, and 24, the pharmacokinetic samples were collected (when possible) following the cognitive and functional assessments. At weeks 10, 16, and 22, the pharmacokinetic samples were collected at any time during the visit.

The primary exploratory analyses for the AI substudy were performed for all randomized subjects who received the study drug at the 10 designated US sites, and included data through week 24. Daily adherence data captured by the AI platform were summarized by week (7-day intervals). The protocol-planned measure for adherence used pill count data based on the returned blister packs. The main adherence measures for the analyses in this paper were based on scheduled pharmacokinetic sampling results and AI platform-measured parameters. A subject on ABT-126 was said to be adherent for a given week based on pharmacokinetics, if the subject’s pharmacokinetic sample taken during that week had measureable study drug concentration (ie, concentration above the lower limit of quantification [LLOQ]). A subject’s AI platform adherence rate for a given week was defined as the number of doses captured by the AI platform relative to the number of planned doses for that week. Additionally, each subject had cumulative pharmacokinetic and AI platform adherence rates calculated for each study week, based on data from that week and previous weeks with nonmissing data. No planned sample size or power calculations were performed for these post hoc exploratory analyses. The analyses are exploratory in nature, and the reported results need to be interpreted descriptively to generate future hypotheses.

## Results

In the M10-855 study, a total of 431 adult subjects with schizophrenia (placebo, n=144; 25 mg ABT‑126, n=66; 50 mg ABT-126, n=151; 75 mg ABT-126, n=70) were randomized and received at least 1 dose of study drug or placebo. Subjects remained on their baseline antipsychotic treatment regimen during the study. Ten of the 31 US sites participated in the AI substudy. A total of 75 subjects were enrolled at these sites (Table 1)). Of these 75 subjects, 53 were monitored with the AI platform. Of these 53 subjects, 12 were grandfathered into the substudy after 6-to-20 weeks of daily administrations of the study drug prior to AI platform monitoring. Among the subjects who used the AI platform, the following completed the study: placebo, 8 of 15 subjects (53%); 25 mg ABT-126, 7 of 8 subjects (88%); 50 mg ABT-126, 12 of 19 subjects (63%); and 75 mg ABT-126, 9 of 11 subjects (82%). All 22 subjects who chose to be monitored by mDOT 3 times per week (per protocol) completed the study (placebo, n=7; 25 mg ABT-126, n=6; 50 mg ABT‑126, n=4; 75 mg ABT-126, n=5). The early discontinuation rate in the AI group (17 of 53 subjects, 32%) was similar to the early discontinuation rate at the 21 US sites not participating in the AI substudy (36 of 135 subjects, 26.7%).

**Table 1:** Subject disposition (AI substudy). AI: artificial intelligence; mDOT: modified directly observed therapy; mg: milligrams.

Parameter		Dose of ABT-126				
		Placebo	25 mg	50 mg	75 mg	Overall
**All subjects participating in the AI substudy, N**		22	14	23	16	75
	Completed study, n (%)	15 (68%)	13 (93%)	16 (70%)	14 (88%)	58 (77%)
	Withdrawn, n (%)	7 (32%)	1 (7%)	7 (30%)	2 (13%)	17 (23%)
**Subjects monitored using the AI platform, N**		15	8	19	11	53
	Completed study, n (%)	8 (53%)	7 (88%)	12 (63%)	9 (82%)	36 (68%)
	Withdrawn, n (%)	7 (47%)	1 (13%)	7 (37%)	2 (18%)	17 (32%)
	Suspicious, n (%)	6 (40%)	4 (50%)	9 (47%)	0 (0%)	19 (36%)
	Grandfathered, n (%)	3 (20%)	2 (25%)	3 (16%)	4 (36%)	12 (23%)
**Subjects monitored using mDOT, N**		7	6	4	5	22
	Completed study, n (%)	7 (100%)	6 (100%)	4 (100%)	5 (100%)	22 (100%)
	Withdrawn, n (%)	0 (0%)	0 (0%)	0 (0%)	0 (0%)	0 (0%)

Suspicious subjects were those flagged by the AI platform as having dosing parameters outside of normal activity. Grandfathered subjects were those enrolled in the study before the option to use the AI platform was introduced.

For all subjects in the AI substudy (n=75), the mean age was 45.9 years (standard deviation [SD] 10.86) and 55% (41/75) of the subjects were male (Table 2). Overall, 52% (39/75) of subjects were black, 41% (31/75) were white, 5% (4/75) were Asian, and 1% (1/75) were Hawaiian. Subject demographics in the AI substudy were similar to those at all US sites (n=210) and mean age was 45.1 years (SD 11.20), 59% of subjects were male, and the majority of subjects were black (57%). In the AI substudy, the treatment groups were reasonably balanced with respect to age, sex, and race, with the exception of a higher percentage of white subjects and lower percentage of black subjects in the 75 mg ABT‑126 group compared with the placebo, 25 mg, and 50 mg ABT‑126 groups.

**Table 2:** Demographic characteristics (AI substudy). mg: milligrams.

Variable		Dose of ABT-126				
		Placebo (N=22)	25 mg (N=14)	50 mg (N=23)	75 mg (N=16)	Overall (N=75)
Age, years	Mean	47.6	45.6	45.0	45.3	45.9
	Standard deviation	7.87	9.52	11.77	14.40	10.86
	Median	47.5	47.5	49.0	48.5	48.0
	Minimum, Maximum	32, 64	29, 62	21, 63	20, 65	20, 65
Sex, n (%)	Female	9 (41%)	8 (57%)	10 (44%)	7 (44%)	34 (45%)
	Male	13 (59%)	6 (43%)	13 (57%)	9 (56%)	41 (55%)
Race, n (%)	Asian	1 (5%)	0 (0%)	2 (9%)	1 (6%)	4 (5%)
	Black	15 (68%)	8 (57%)	12 (52%)	4 (25%)	39 (52%)
	Hawaiian	0 (0%)	1 (7%)	0 (0%)	0 (0%)	1 (1%)
	White	6 (27%)	5 (36%)	9 (39%)	11 (69%)	31 (41%)

**Figure 2:**
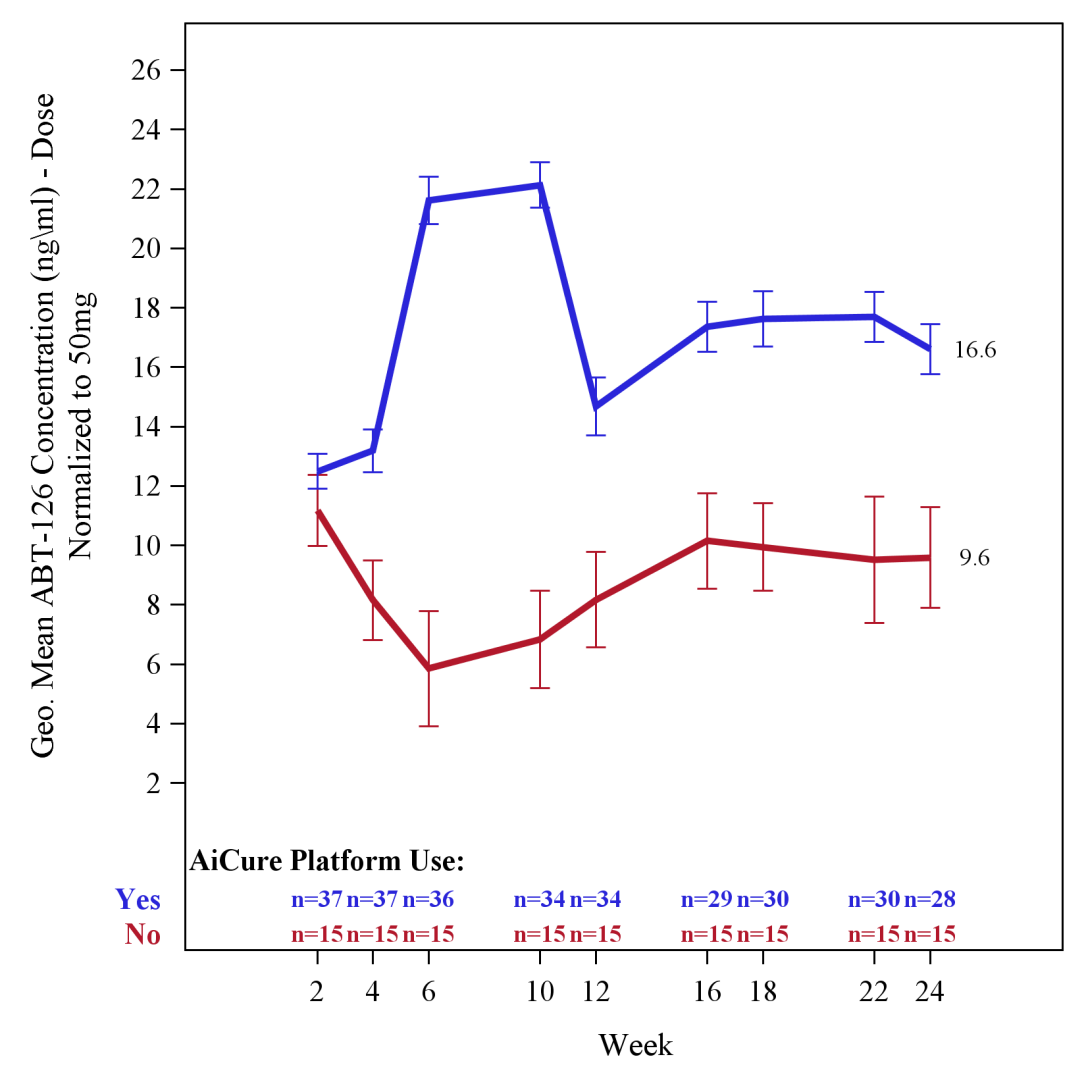
Geometric mean ABT-126 plasma concentrations, normalized to the 50 milligram dose, for subjects who participated in the adherence substudy stratified by artificial intelligence platform versus modified directly observed therapy use. Error bars indicate mean with standard errors. ng/mL: nanograms/milliliter; mg: milligram.

Subjects who were monitored by the AI platform, received ABT-126 (25, 50, or 75 mg), and had available pharmacokinetic data had geometric mean ABT‑126 drug levels (normalized to the 50 mg dose) that were higher at each time point evaluated through week 24 compared with subjects monitored by mDOT (Figure 2). At week 24, the geometric mean drug level, normalized to the 50 mg dose, was 16.6 nanograms/milliliter (ng/mL; SD 4.42) for subjects using the AI platform compared with 9.6 ng/mL (SD 6.56) for subjects monitored by mDOT. The analysis set consisted of all subjects who received any dose of ABT-126 at the participating US sites and had available pharmacokinetic data. Visits were based on a categorization of collection-day data into weekly windows.

**Figure 3:**
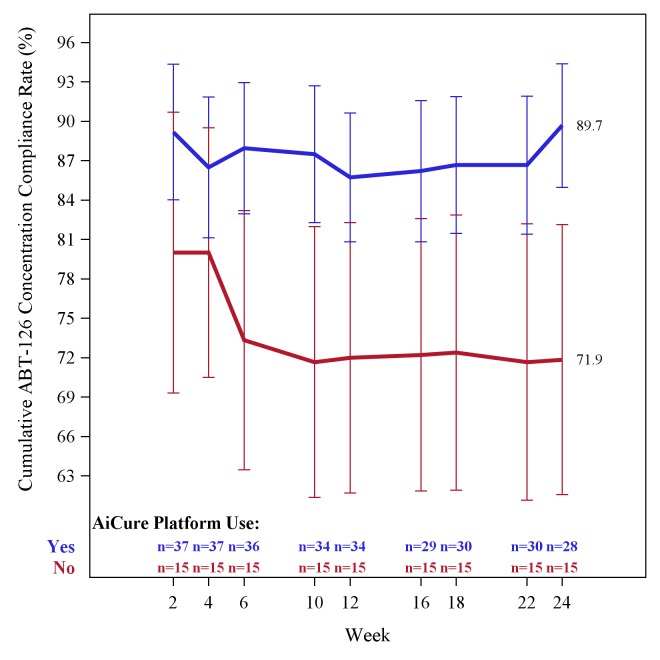
Cumulative adherence based on study drug (ABT-126) concentration.

Based on an analysis of subjects who received ABT-126 at the AI substudy sites and had available drug concentration data, cumulative pharmacokinetic adherence was higher from week 2 through week 24 for subjects monitored using the AI platform compared with subjects monitored using mDOT (Figure 3). The mean cumulative pharmacokinetic adherence over 24 weeks was 89.7% (SD 24.9) for subjects receiving ABT-126 and monitored using the AI platform (n=28) compared with 71.9% (SD 39.8) for subjects receiving ABT-126 and monitored by mDOT (n=15). The difference was 17.9% (95% CI -2 to 37.7; *P*=.08). Subjects (n=69) at the 21 US sites not participating in the AI substudy had cumulative pharmacokinetic adherence over 24 weeks of 78.1% (SD 29.7). The analysis set consisted of all subjects who received any dose of ABT-126 at US sites selected to use the AI Platform and who had available pharmacokinetic data. Visits were based on a categorization of collection day data into weekly windows. Pharmacokinetic adherence was defined as ABT-126 levels greater than the LLOQ (0.7 ng/mL). Cumulative results were based on data from current and previous visits with nonmissing data.

A total of 19 subjects (19/53, 35.8%; 13 subjects on active drug [including 4 subjects in the 25 mg ABT-126 group and 9 subjects in the 50 mg ABT‑126 group] and 6 subjects on placebo) were flagged as having suspicious drug administration behavior by the AI platform (Table 1). The generation of the platform used in the study utilized manual review of deidentified video data to identify suspicious behaviors (leaning out of the field of view, tampering with the drug, spitting out the drug, hand to mouth gestures, and turning the device away). Seven of 13 subjects (54%) had at least 1 pharmacokinetic sample showing a drug concentration of zero or below LLOQ, or did not complete the trial. At week 24, mean cumulative pharmacokinetic adherences for suspicious subjects using the AI platform (n=10), for nonsuspicious subjects using the AI platform (n=18), and for subjects monitored using mDOT (n=15) were 78.9% (SD 36.8), 95.7% (SD 12.7), and 71.9% (SD 39.8), respectively. At week 24, mean cumulative pharmacokinetic adherence for subjects grandfathered into the substudy and using the AI platform (n=7) was lower (81.0%, SD 37.8) compared to those who began the study with the AI platform (n=21; 92.6%, SD 19.4).

The average cumulative dose adherence measured by the AI platform through week 24 was 80%. Of note, concordance was not demonstrated between the AI platform and pharmacokinetic cumulative adherence rates, giving a Pearson’s correlation of r=0.33 (95%CI -0.12 to 0.65). At the AI substudy sites, the mean percentage of subjects with adherence >70%, as measured by review of returned study drug blister cards, was >90% at all study visits evaluated (weeks 2, 4, 6, 10, 12, 16, 18, 22, and 24) for each treatment group (including subjects monitored with the AI platform and those monitored by mDOT).

Subjects received 3 study drug capsules per daily dose, for a total of 21 capsules per week. The mean total capsules per week based on visual confirmation of ingestion by the AI platform ranged from 14.6 to 18 and the total mean missed/skipped capsules per week ranged from 1 to 3 for subjects receiving any dose of study drug (placebo or ABT-126) who were monitored using the AI platform. Overall, the mean time required to take a capsule (placebo or active drug) while being monitored with the AI platform ranged from 40.3 to 70.6 seconds from weeks 1 to 24. The average time taken per dose of study drug (3 capsules; placebo or active drug) throughout the study was 3 minutes.

## Discussion

Electronic monitoring of medication adherence [23] has highlighted the value of obtaining real‑time dosing data for better informed pharmacokinetic, pharmacodynamic, and efficacy analyses, and for treatments requiring close monitoring [12,24]. Although the accuracy of electronic monitoring has been validated based on observed dosing [25], most studies have not evaluated electronic monitoring using drug levels in the blood; sensitivity and specificity are typically measured against biomarkers such as detected HIV viral load [26-28] or adherence is compared to less reliable measures, such as pill counts or patient self-reports [16].

Other monitoring methods using ingestible sensor technology or breathalyzer monitoring have demonstrated the accuracy of these methods based on adherence markers or plasma drug concentrations collected during observed dosing [29,30]. However, usability outside of controlled settings has not been robustly assessed [29,30]. Evaluations of new monitoring methods should include effects on adherence, concordance with drug concentration measurements in ambulatory settings, and ease of adoption.

### Principal Results

In the present study, the AI platform was introduced into 10 of 31 US study sites participating in a Phase 2 study of ABT-126 in subjects with schizophrenia to augment standard and more intensive adherence assessments (eg, pill counts and mDOT), and to evaluate and test feasibility of the AI platform in this setting. Adherence measures (AI platform vs mDOT) were compared with drug concentration measurements to build a framework for evaluating the effectiveness of the AI platform in measuring drug ingestion and promotion of treatment adherence. Subjects monitored using the AI platform appeared to be more adherent to study drug dosing compared to subjects receiving mDOT 3 times per week, based on plasma drug levels. The differences detected in study drug concentrations might have been more pronounced had a measure such as mDOT (which helps support optimal adherence [21]) not been the comparator in this study, or had a drug with a shorter half-life than ABT-126 been evaluated (the plasma concentrations of which would have been more sensitive to missed doses).

Lack of concordance between the AI platform and pharmacokinetic cumulative adherence rates, giving a Pearson’s correlation of r=0.33 (95%CI -0.12 to 0.65), may be explained by the following three considerations: (1) 9 patients receiving active study drug were grandfathered into the substudy, each of whom had pharmacokinetic samples with no corresponding AI platform adherence data; (2) the AI platform dataset contained a maximum of 540 data points for each subject, compared to a maximum of 9 data points for each subject in the pharmacokinetic dataset; and (3) the AI platform dataset included doses that were missed, skipped, or self-reported, highlighting suboptimal adherence that may not have been captured in the pharmacokinetic dataset.

Cumulative adherence, measured by study drug concentrations above the LLOQ, appeared to be higher through 24 weeks for subjects monitored using the AI platform (89.7%) compared with subjects monitored using mDOT (71.9%). The difference was 17.9% (95% CI -2 to 37.7; *P*=.08).

Subjects monitored using the AI platform demonstrated a percentage change in adherence of 25% over the mDOT group. The study drug concentration among the mDOT group (71.9%) is consistent with the McCann et al study, which showed nonadherence rates of 12.8-39.2% [2]. Deceptively removing pills to feign higher adherence leads to the low rates of nonadherence typically measured by pill count, as seen in the aforementioned study (0.0-5.1% [2]) and in the present study (0-3%). The discrepancy between the adherence rates reported by pill count and those measured through pharmacokinetic sampling is substantial. Within the AI group, subjects who were identified as suspicious had lower cumulative pharmacokinetic adherence at week 24 compared to subjects who were not identified as suspicious (78.9% vs 95.7%). Subjects who were grandfathered into the substudy had lower cumulative pharmacokinetic adherence at week 24 compared to subjects who were not grandfathered into the substudy (81.0% vs 92.6%). Excluding these first 2 groups might have led to higher cumulative adherence in the AI group.

The AI platform was successfully utilized in this multicenter study among cognitively impaired subjects with schizophrenia. Subjects were able to use the technology successfully for up to 6 months in an ambulatory setting. Acceptance of mobile technology has received little attention in this patient population and has primarily relied on the use of mobile devices for patient self‑assessment, and as psychoeducational tools [31,32].

### Limitations

The principal limitation of this substudy was the lack of randomization; subjects were allowed to choose between the AI platform and mDOT. Of the 75 subjects at the 10 sites, 22 self-selected mDOT, possibly adding bias to the study results. However, it is worth noting that 17 of the 22 subjects had already started the study prior to the introduction of the AI platform, or were already receiving DOT at board and care facilities. A second limitation was the small sample size of subjects randomized to active drug (AI platform, n=38; mDOT, n=15). A third limitation was the inconsistent use of mDOT. Although mDOT usage was suggested at all US sites, not all subjects received direct observation 3 times per week. A fourth limitation was the use of the plasma concentrations in the analyses without regard to the collection time relative to the recorded last dose. The impact of this variable on the conclusions is believed to be negligible since the threshold for declaring lack of adherence was a concentration below the LLOQ, which is a conservative criterion that should not be very sensitive to collection time relative to dosing time variations.

### Conclusions

Extensive evidence of nonadherence in clinical trials, which includes behaviors such as removing pills from blister cards or bottles while reporting high adherence, can undermine trial results by providing false data and preclude true assessments of efficacy and safety [3]. AI platforms have the potential to increase adherence, identify poor‑performing subjects, and improve data quality. Detailed dosing patterns based on confirmed ingestions allow for real‑time intervention to further improve adherence rates and subject retention. In clinical practice, where poor adherence to antipsychotic treatment is linked to increased hospitalization rates, the availability of real-time data could allow for quicker and more effective interventions, potentially improving outcomes and reducing relapses [33]. In clinical research, this technology might be useful to predict future behavior during placebo lead-in periods in clinical studies by allowing for early detection and intervention [3,34]. Such technology can also help researchers understand the response patterns among patients in trials and terminate the development of ineffective drugs with confidence, leading to improved decision-making and accelerated clinical trial results. The use of AI to visually confirm medication ingestion is a valid contribution to the armamentarium of tools that could help reduce uncertainties and costs associated with high rates of nonadherence in clinical trials and real-world settings.

## Abbreviations

AI: artificial intelligence
DOT: directly observed therapy
EMP: electronic monitoring packaging
HIV: human immunodeficiency virus
LLOQ: lower limit of quantification
mDOT: modified directly observed therapy
mg: milligram
mL: milliliter
ng: nanogram
SD: standard deviation
